# Supplementary material for: SpikePropamine: Differentiable Plasticity in Spiking Neural Networks
Source: Front Neurorobot. 2021 Sep 22;15:629210. doi: 10.3389/fnbot.2021.629210 (PMC8493296; doi:10.3389/fnbot.2021.629210)
Supplement: Supplementary file 1 [file Data_Sheet_1.pdf]

Supplementary Materials for SpikePropamine: Differentiable Plasticity in Spiking Neural Networks

Samuel Schmidgall, Julia Ashkanazy, Wallace Lawson, Joe Hays *

U.S. Naval Research Laboratory, Washington D.C., United States

Correspondence*:
Joe Hays
joe.hays@nrl.navy.mil

1 SUPPLEMENTARY

1.1 SLAYER Implementation

The original code for SLAYER[1], specifically the PyTorch version, was developed in CUDA to support a specific type of learning where the information regarding the temporal dataset was required to be known a-priori. In this assumed domain, a single network output was attributed to a single interaction episode, where the membrane potential was reset between these episodes. These dynamics do not support the type of flexible interactions required by RL problems, since often the interaction boundary is variable and actions must be evaluated many times before the membrane potential is reset. Toward this effort, we modified the SLAYER PyTorch library to accommodate RL applications at the low-level as well as having developed a complimentary RL framework using PPO to fit our needs. The modified SLAYER and PPO framework supports learning on the SRM model described in our experiments.

1.2 Input Population Representation

Specified here is the input population representation used for the high-dimensional locomotion experiment:

$$\begin{aligned} &\forall m \in \{0, 1, ..., P_{dim} - 1\}, \forall \xi \in \{0, 1, ..., P_{num} - 1\}, \\ &\Omega_{\xi,m} = P_{\xi,min}(m - P_{num}) - P_{\xi,max}(P_{num} - m) \end{aligned} \tag{1}$$

$$Pr[s^{(0)}_{\xi,m} = 1] = max(\vartheta_{min}, min(exp(-15(\Omega_{\xi,m} - x_\xi)^2), 1)). \tag{2}$$

Here, the initial number of input sub-populations are defined as $P_{num} \in \mathbb{N}$ and indexed by $\xi \in \mathbb{N}$, which correspond to the number of floating point values in the pre-converted input vector. Additionally, an initial neuron population size, $P_{dim} \in \mathbb{N}$, is specified to represent each floating point input and indexed by $m \in \mathbb{N}$. Using this population, the minimum and maximum state values, $P_{\xi,max} \in \mathbb{R}$ and $P_{\xi,min} \in \mathbb{R}$, are represented by the first and last neuron in the population, and each neuron in-between is an intermediate value, $\Omega_{\xi,m} \in \mathbb{R}$, linearly distributed based on the population size (1). Once the place cells are appropriately represented and an incoming stimulus, x_ξ, is present, the probability of spiking for each neuron is assigned using an exponentially decaying probability distribution (2). The exponential reaches its maximum value around the place neuron, $s^{(0)}_{\xi,m}$, that most closely resembles the incoming stimuli x_ξ, with each subsequent neuron in the population having a likelihood representative of the distance from this initial cell $(\Omega_{\xi,m} - x_\xi)^2$.

[1] https://github.com/bamsumit/slayerPytorch

Additionally, a pre-defined spike probability, $\vartheta_{min} \in \mathbb{R}$, is assigned globally to each neuron independent from the distance, which was experimentally shown to improve performance on this task. We note here that the spike-activity produced by (1-2), $s^{(0)}_{\xi,m}$, directly corresponds to the spike input defined in Section 2.

1.3 Action Population Representation

$$A_p = \frac{1}{T}\sum_{t=0}^{T}(\sum_{n=0}^{N} w_n S_n(t)) \tag{3}$$

where $A_p \in \mathbb{R}$ denotes the action produced over a sub-population $p \in \mathbb{N}$. The ordered-tuple of sub-population actions $A = (A_0, A_1, ..., A_d)$ produces the final action for each actuated joint p, where $d \in \mathbb{N}$ is equal to the number of actuated joints. The variable $T \in \mathbb{N}$ represents the discrete time interval duration from which the action is averaged over. Additionally, $N \in \mathbb{N}$ represents the total number of neurons in the action sub-population. $S_n(t) \in \{0, 1\}$ is the binary spike output of neuron n at time t, and $w_n \in \mathbb{R}$ weights the spike. In this experiment, $w_n = 1$ for half of the population $n < \frac{N}{2}$, and otherwise $w_n = -1$. This produces a natural mapping over the interval $[-1, 1]$ for each sub-population A_p, where simple shifting and scaling enables representation over arbitrary intervals.

1.4 Hardware

Installed Physical Memory (RAM): 64.0 GB
Graphics Card: NVIDIA Quadro P2000, 5 GB GDDR5, 1024 CUDA cores
Processor: Intel Xeon W-2125 CPU 4.00GHz, 4 Core(s), 8 Logical Processor(s)

1.5 Neuron Model Hyperparameters

Provided is a list of the neuron model hyperparameters used for the experiments in this paper. The SRMALPHA neuron type is originally described in the SLAYER code repository. We note that in practice, the behavior of the system acts independent of the defined metric units.

Hyperparameter Table	
Neuron Type	SRMALPHA
Threshold	10 (mV)
Neuron time constant	10 (ms)
Network integration time	1 (ms)
Refractory time constant	2 (ms)
Neuron relative refractory response scaling	2
Spike function derivative time constant	1
Spike function derivative scale factor	1

1.6 Cue-Association Training Details

Hyperparameter Table	
Cue Labels	2
Total Presented Cues	7
Cue Presentation Time	25 (ms)
Noise Population Neurons	10
Cue Population Neurons	10×3
Horizon	500 (Steps)
Discount (λ)	0.99
Adam Timestep	$5 \times 10^{-4} \times \alpha$
Cue Spike Event Prob	0.75
Cue Spike Rest Prob	0.05
Noise Spike Rest Prob	0.2
Cue Spike Event Prob (Noisy)	0.65
Cue Spike Rest Prob (Noisy)	0.25
Noise Spike Rest Prob (Noisy)	0.4
Rest Period	$r \sim \{45, 75, 105\}$(ms)

1.7 Cue-Association: Neuronal Activity

Here we provide additional insights for the neuronal activity during the cue-association task. The highest performing network from Experiment 1, NDP-BCM, is used for analysis.

Four cue-association cases are considered: a typical random sensory input sequence, all left cues, all right cues, and one with no sensory cues except for spike-noise. Interestingly, each of the output modulatory signal graphs exhibit a loose symmetry (Figures 6-9). The hidden modulatory signals follow a similar dynamic pattern. In the first three sequences (Figures 6-8), the weight values tend to both potentiate and depress during the cue presentation, with a stronger emphasis on negatively valued weights. The weights then seem to depress significantly during the period in which cues are absent only to potentiate again in the presence of the decision cue. However, in the fourth sequence (Figure 9), when no signals are present, the weight values do not meaningfully potentiate, and also do not depress beyond an initial range of values. The hidden modulatory activity in this case does not follow a pattern resembling the first three sequences, and seems to develop without much pattern at all. While the output modulatory signals do still exhibit a symmetry, it is the only scenario in which the signals strictly diverge from each other. It is evident that the plasticity and neuromodulatory signals have a strong effect on self-organization during the cue period, with a large population of weights undergoing drastic changes from experience-dependent activity.

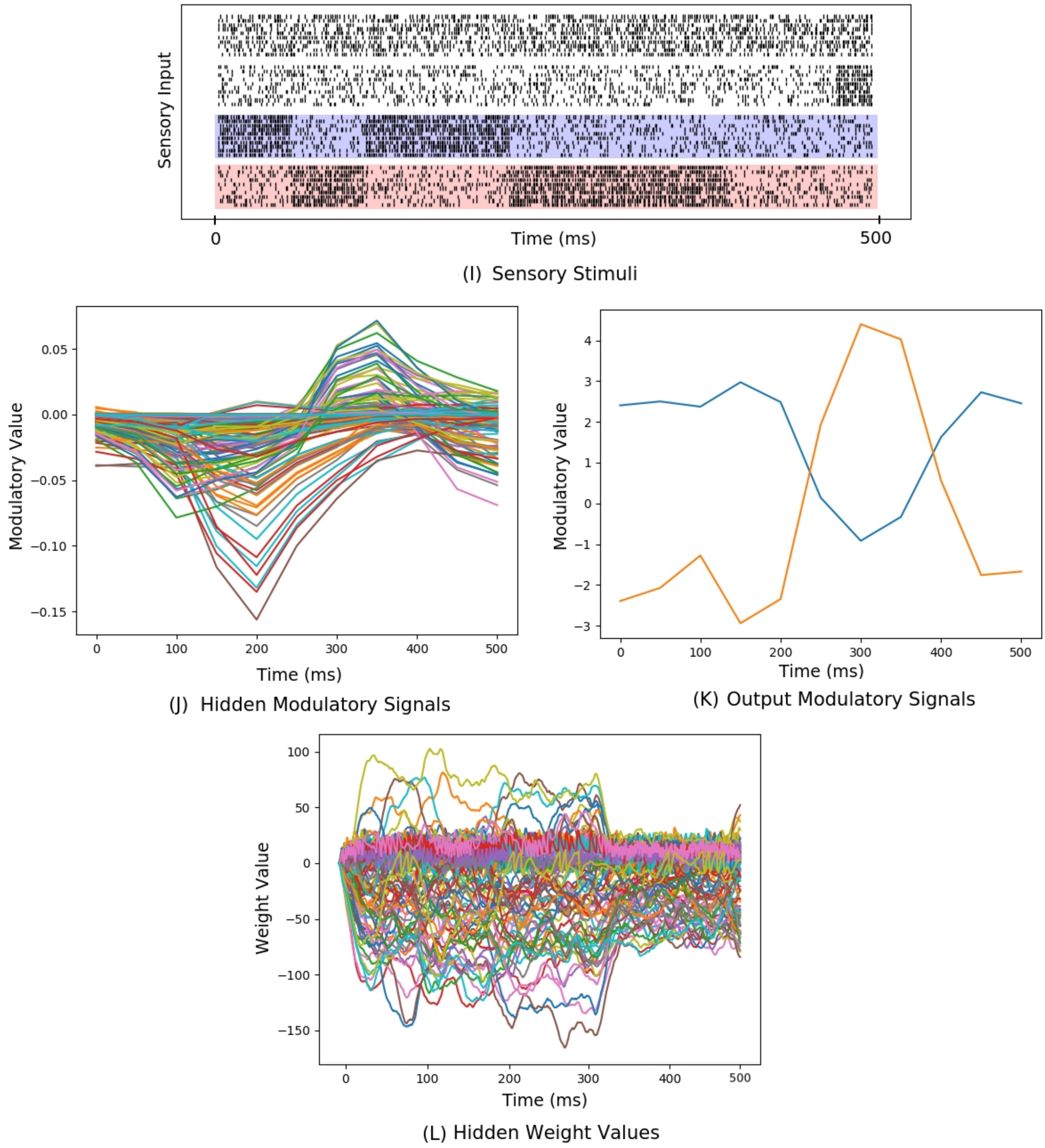

Figure 1. Typical Cue Sequence

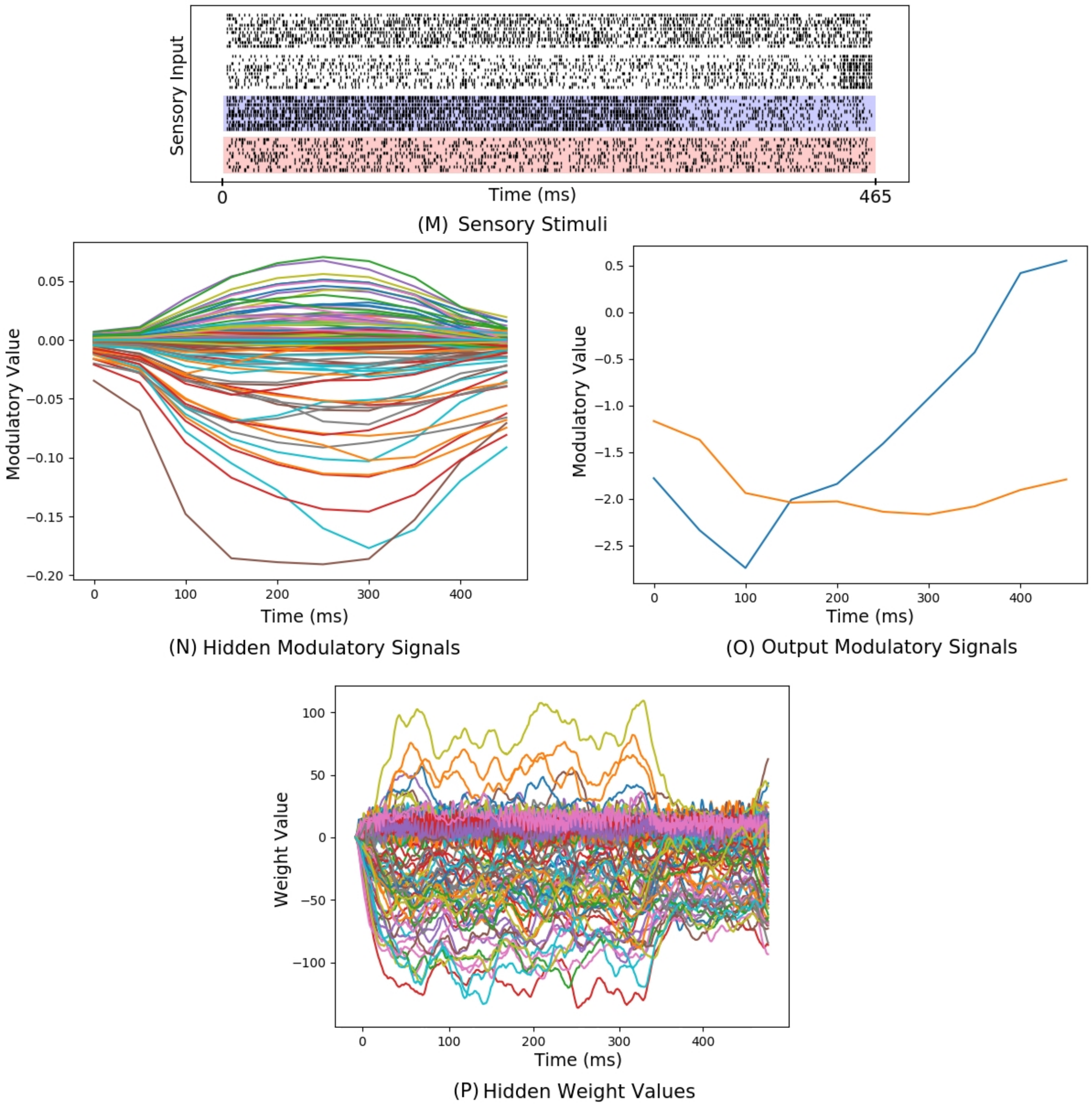

Figure 2. Only Left Cues (Blue)

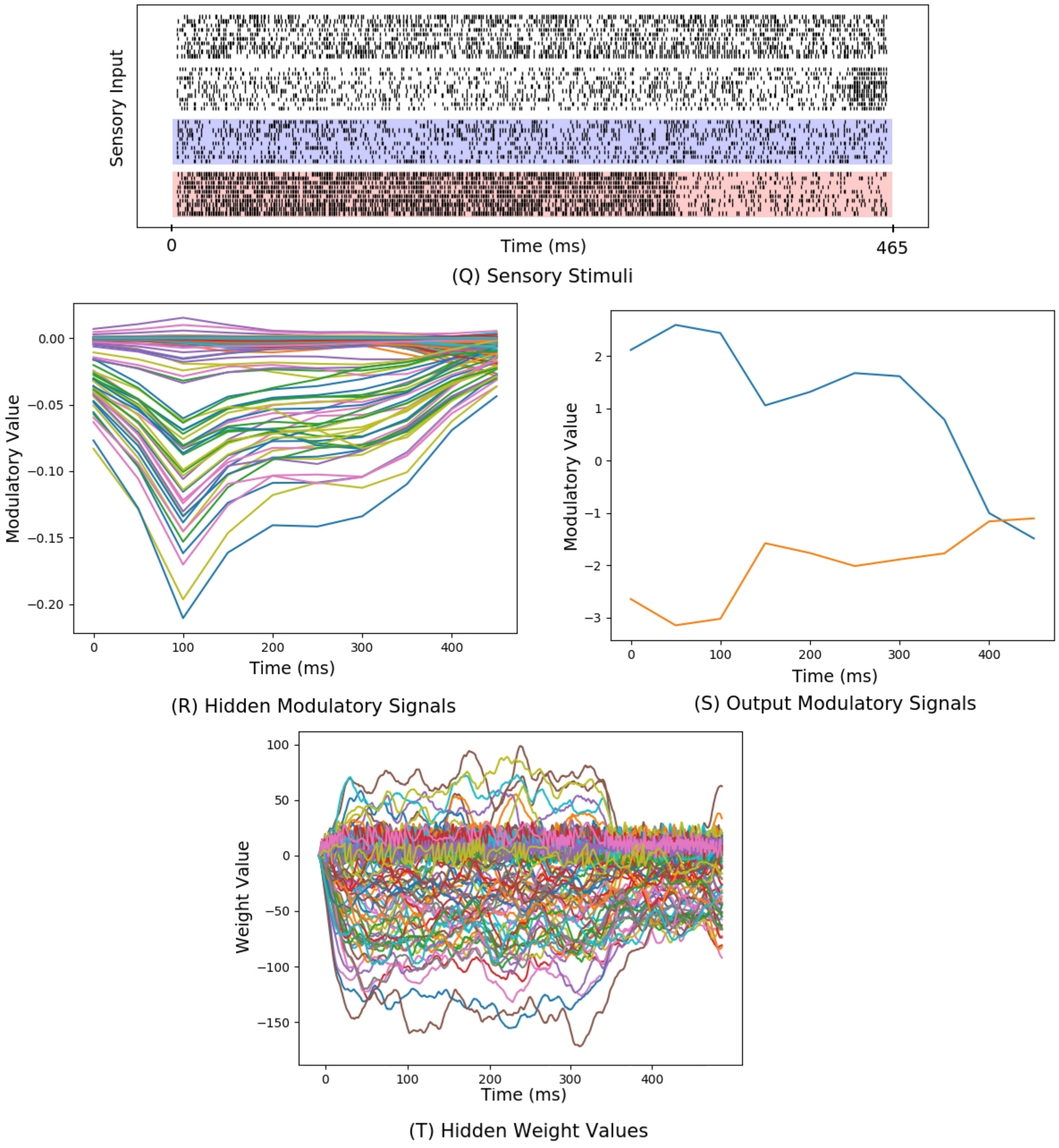

Figure 3. Only Right Cues (Red)

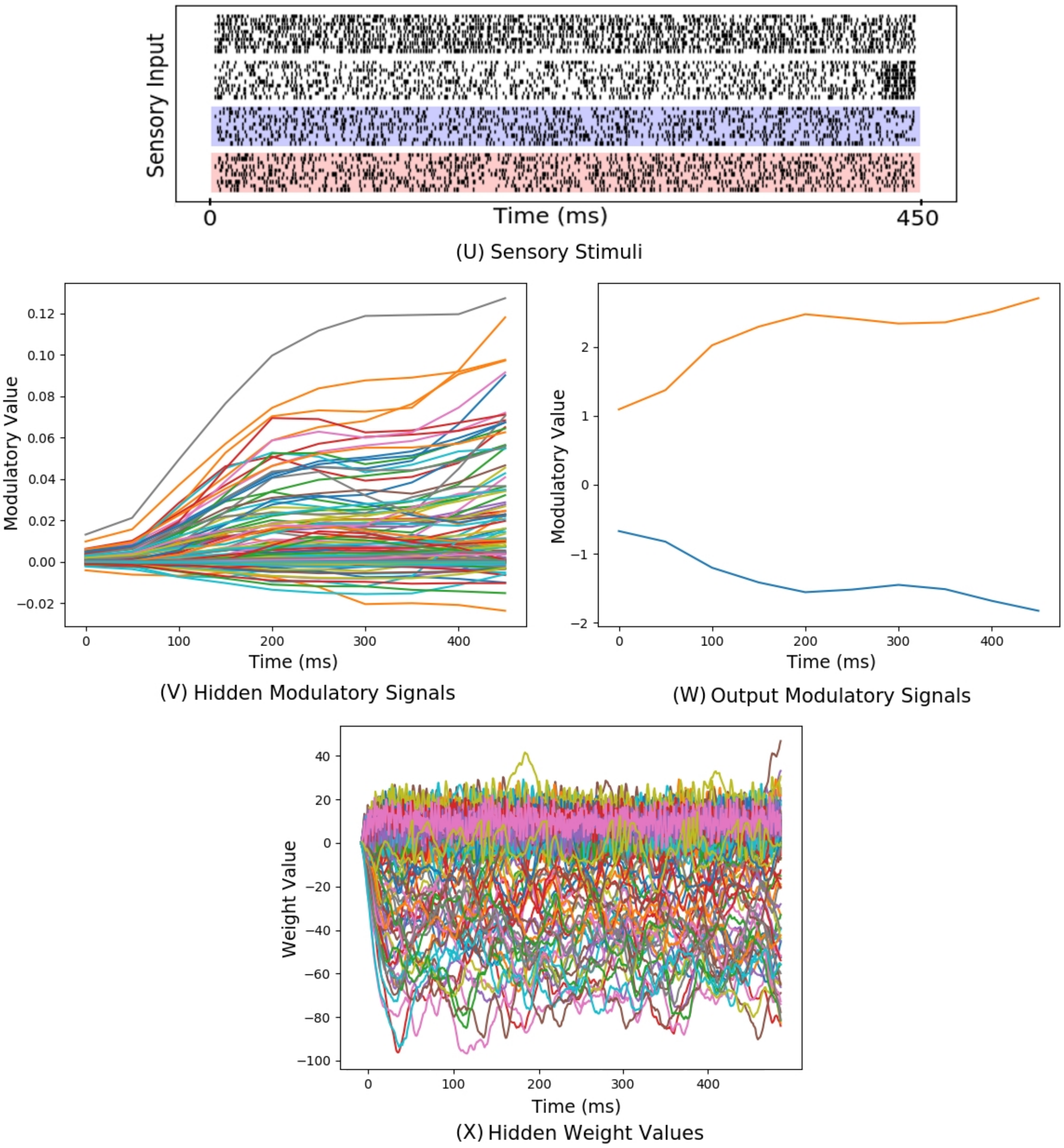

Figure 4. No Sensory Input

1.8 Half-Cheetah Training Details

To compute the advantage for the Proximal Policy Optimization gradient update, Generalized Advantage Estimation (GAE) is used.

Hyperparameter Table	
Horizon	3000 (Steps)
PPO Epochs	10
Adam Timestep	$5 \times 10^{-4} \times \alpha$
Discount (γ)	0.99
GAE lambda (λ)	0.97
PPO Updates	1500
Random Spike Prob (ϑ_{min})	0.05
Action Integration Interval (T)	50 (ms)

1.9 High-dimensional Robotic Locomotion: Neuronal Activity

Here we provide additional insights into the internal neuronal activity for the robotic locomotion task. On this task, results are shown using with the highest-performing network from Experiment 1, NDP-Oja's.

(Figure 10 (A-D)) shows the modulatory behavior in the first hidden layer using the same network for two scenarios: when the robot is flipped on it's back (Figure 10 (A)), and when the robot is successfully performing locomotion (Figure 10 (B)). In both of these cases the action output layer is amplified by $\pm\mathcal{N}(0, 30)\%$ action noise at each timestep. In the case of successful locomotion (Figure 10 (B)), it is observed that each modulatory signal oscillates within a set region determined within 50 timesteps of the simulation. The majority of signals cluster around 0, however some signals are distributed within the range of ± 4. When deprived of sensory stimuli in the flipped scenario (Figure 10 (A)) the signals still seem to display a similar distribution, however they do not exhibit nearly any oscillations. Additionally, these signals are notably larger than the hidden layer signals in the cue-association task, and do not display the same characteristic movement. Perhaps this noisy distribution plays a critical role in the adaptive behavior observed in Experiment 2.

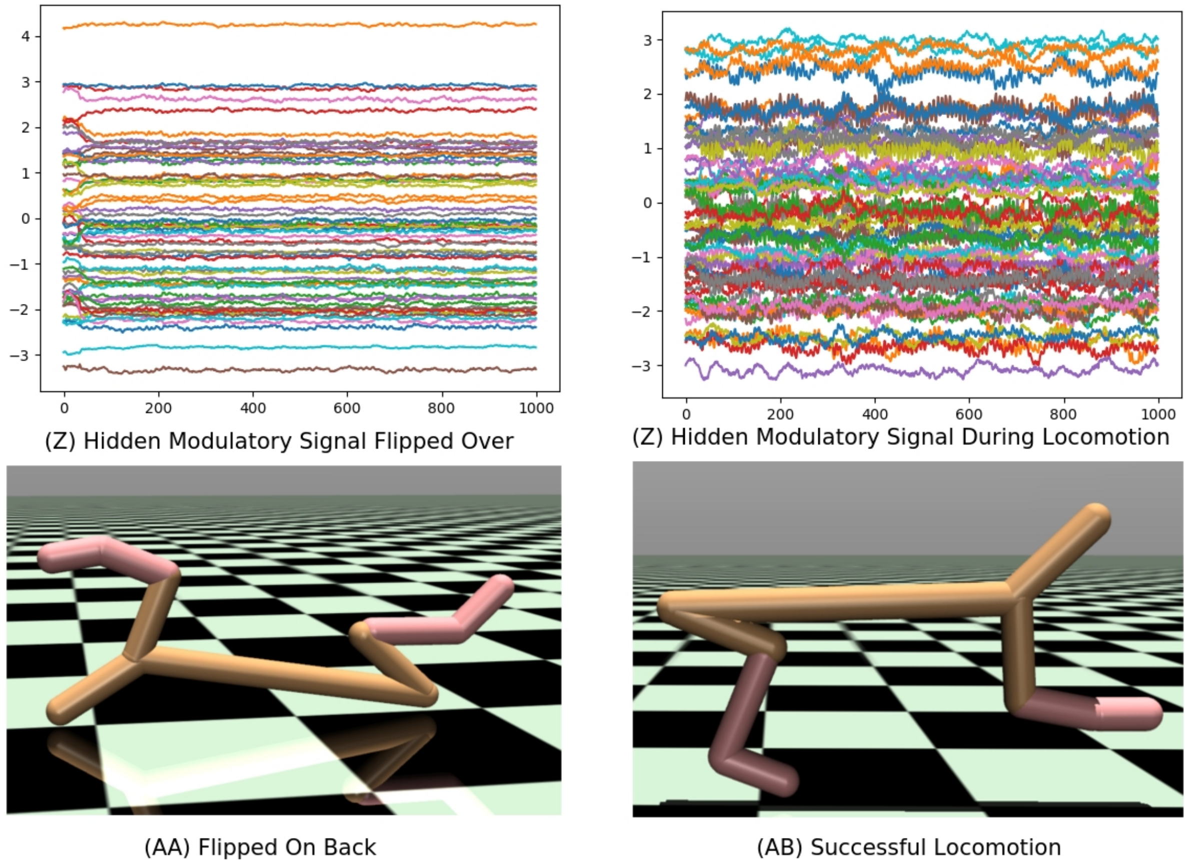

Figure 5. Neuromodulatory activity in hidden layer with $\pm\mathcal{N}(0, 30)\%$ action noise when (AA) robot is flipped on back and (AB) successfully solves locomotion task. While the neuromodulatory signals across neurons seem to remain within a consistent activity region, the oscillatory behavior seems to play a role in sensory processing since, when deprived of sensory stimuli (flipped on it's back), the signals drastically reduce any change in activity.
